# Hyperglycemia-related central pontine demyelinization after a binge-eating attack in a patient with type-2 diabetes: a case report

**DOI:** 10.1186/s12902-018-0245-3

**Published:** 2018-03-12

**Authors:** Rainer U. Pliquett, Arno Noll, Richard Ibe, Alexandra Katz, Charlotte Ackmann, Alexandra Schreiber, Matthias Girndt

**Affiliations:** 1 Department of Internal Medicine II, Martin-Luther-University Halle-Wittenberg, Ernst-Grube-Str. 40, 06120 Halle (Saale), Germany; 2 Department of Radiology, Martin-Luther-University Halle-Wittenberg, Halle (Saale), Germany; 3 Department of Neurology, Martin-Luther-University Halle-Wittenberg, Halle (Saale), Germany

**Keywords:** Pontine demyelinization, Hyperglycemia, Compliance

## Abstract

**Background:**

Here, we report a case of central pontine demyelinization in a type-2 diabetes patient with hyperglycemia after a binge-eating attack in the absence of a relevant hyponatremia.

**Case presentation:**

A 55-year-old, male type-2 diabetic patient with liver cirrhosis stage Child-Pugh B was admitted due to dysmetria of his right arm, gait disturbance, dizziness, vertigo, and polyuria, polydipsia after a binge-eating attack of sweets (a whole fruit cake and 2 Liters of soft drinks). A recently initiated insulin therapy had been discontinued for 8 months. A serum glucose measurement obtained 5 days prior to hospitalisation was 38.5 mmol/l (694 mg/dl). The patient graved for sweets since stopping alcohol consumption 8 months earlier. On admission, venous-blood glucose was 29.1 mmol/l (523.8 mg/dl), glycated hemoglobin was 168.0 mmol/mol or 17.6%. No supplementation of sodium chloride was reported. Laboratory exams revealed an elevated serum ammonia level (127.1 μmol/l), rendering a hepatic encephalopathy very likely. After initiation of insulin therapy, capillary glucose normalized, and serum sodium rose from 133 on admission to 144 mmol/l during the hospital stay. In retrospect, the mild hyponatremia on admission was classified as pseudohyponatremia due to hyperglycemia. The patient had an insulin resistance (HOMA-IR 7.8 (normal range < 2.5)). A T2-weighted magnetic resonance imaging (MRI) of the head and a cranial computed tomography scan were obtained demonstrating a symmetric central pontine demyelinization. After 26 days in hospital, the patient was discharged with an inkretin-mimetic therapy (dulaglutide SC, 1.5 mg/week) and an intensified conventional insulin therapy (insulin aspart: 14 units/d in euglycemia, insulin glargin 20 units/d).

**Conclusions:**

Central pontine and/or cerebellar myelinolysis can be caused by sudden, severe, and sustained hyperglycemia, especially when another risk factor (in this case, liver cirrhosis) is present. Functional neurological deficits in the context of hyperglycemia should prompt for the consideration of this differential diagnosis in all diabetes patients.

## Background

Central pontine demyelinization, also referred to as osmotic demyelination syndrome or central pontine myelinolysis, has been reported after increases in serum osmolality due to overcorrection of hyponatremia [1]. As a pathomechanism, an increase in serum osmolality due to a rapid correction of hyponatremia is well recognized [2].

In single cases, hyperglycemia leading to an increased plasma osmolality has been attributed to cause a central pontine demyelinization in type-2 diabetes patients [3, 4] and in a type-1 diabetes patient [5].

This complication also has been reported in a patient with latent autoimmunity diabetes displaying significant glucose fluctuations [6].

Here, we report a case of central pontine demyelinization in a type-2 diabetes patient with a severe hyperglycemia on admission in absence of a clinically relevant hyponatremia.

## Case presentation

A 55-year-old, male type-2 diabetic patient with alcoholic liver cirrhosis was admitted as an emergency due to a suddenly occurring dysmetria, lack of coordination of his right arm, weakness and difficulty to speak since 5 days prior to hospitalization. In addition, gait disturbance, dizziness and vertigo with tendency to fall to the right side, and an intractable pain in both legs occurred 1 day prior to hospitalization. Lastly, he reported a worsening polyuria, polydipsia, and peripheral edema. On admission, the level of consciousness appeared to be normal. Medication included lisinopril (2.5 mg/d), carvedilol (6.25 mg/d), pantoprazole (40 mg/d), pregabaline (75 mg/d), xifaxan (500 mg/d), sodiumhydrogencarbonate (1 g/d) and lactulose solution. Comorbidities included carpal-tunnel syndrome and gonarthrosis.

Six days prior to hospitalization, the patient reported the ingestion of a whole fruit cake (estimated glucose amount: 154 g) and, concurrently, the consumption of 10 soft drinks (2 Liters, estimated sugar content: 212 g according to the ingredients’ list). During this binge-eating attack, the estimated grand total of ingested sugar was 366 g. According to the family physician, the graving for sweets occurred since the patient refrained from alcohol 8 months ago. A serum glucose measurement obtained by the family practitioner 5 days prior to hospitalisation was 38.5 mmol/l (694 mg/dl). On admission, venous-blood glucose was 29.1 mmol/l (523.8 mg/dl), glycated hemoglobin was 168.0 mmol/mol or 17.6% (result as percentage was calculated using the following formula: HbA1C [%] = HbA1C [mmol/mol] * 0,0915 + 2,15) demonstrating a poor glycemic control over the last 6 weeks. Four months prior to admission, glycated hemoglobin still was 62.8 mmol/mol or 7.9%.

On admission, a mild hyponatremia of 133 mmol/l was found. Of note, no hyponatremia had been detected prior to hospitalization. In addition, sodium-chloride supplementation or any other voluntary salt intake were not reported. The concomitant liver cirrhosis (Child-Pugh B; first diagnosis: 28 months earlier) due to alcoholism over 18 years was treated by a transjugular porto-systemic shunt 7 months prior to the index hospitalization. In addition, 8 months prior to index hospitalisation, a subcutaneous insulin regimen (fixed-dose prandial lispro insulin. Cumulative dose: 34 units per day) was initiated for documented hyperglycemic episodes during a hospital stay for decompensated liver cirrhosis. However, at that time, the patient discontinued the insulin therapy after discharge. Outpatient capillary-blood glucose tests, except shortly prior to index hospitalisation, were not performed. A reevaluation of the patient’s capacity to apply insulin therapy at home was not performed neither.

**Fig. 1:**
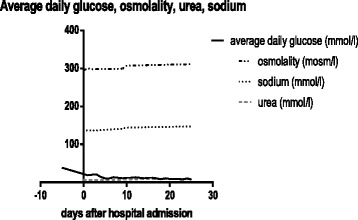
Daily average capillary-blood glucose readings during the hospital stay

Laboratory exams during index hospitalisation revealed elevated serum ammonia levels (127.1 μmol/l on admission), rendering a hepatic encephalopathy very likely. In-hospital blood tests for ethanol were negative. Estimated glomerular filtration rate (eGFR) ranged between 46 and 56 ml/min/1.73m^2^ during the hospital stay, proteinuria was ruled out, thus confirming the preexisting chronic kidney disease stage G3aA1 (kidney disease improving global outcomes (KDIGO) classification [7]). An infection or sepsis were ruled out on admission. After initiation of insulin therapy, capillary-blood glucose normalized (Fig. 1), and serum sodium rose to 144 mmol/l during the hospital stay. In retrospect, the mild hyponatremia on admission was classified as pseudohyponatremia due to hyperglycemia.

The patient was shown to have a state of insulin resistance with an HOMA-IR index of 7.8 (normal range < 2.5 [8]) on the 4th day in hospital (variables used: fasted venous blood glucose: 16 mmol/l, fasted serum insulin: 11 mlU/l).

The serum ammonia level declined under conservative therapy by the 11th day in hospital to 70.5 μmol/l. Liver transaminases and total bilirubin were stable within normal range throughout the hospital stay, however, direct bilirubin was slightly elevated on one occasion (10.1 μmol/l, normal range: < 5). Repeat measurements of alkaline phosphatase and of gamma glutamyl transferase revealed slight elevations (less than 3× of upper normal limit).

A neurological exam was performed on the 9th day in hospital when the neurocognitive performance and the level of consciousness suddenly decreased. No paresis was found. Strümpell sign, a pyramidal sign, was positive, Babinski sign was negative. In addition, dysarthria, and signs of dysmetria, dysdiadochokinesis on both sides and an ataxia both at rest and when walking were found. On the next day, the patient was somnolent. The level of consciousness gradually improved by the 15th day in hospital. By then, all other above-mentioned neurological findings were improving as well.

**Fig. 2:**
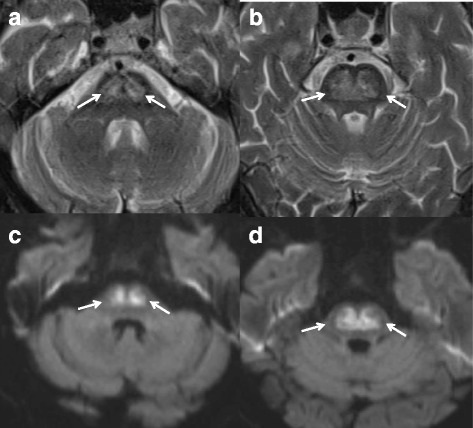
MRI images of the brain (**a** and **b**: T2-weighted, **c** and **d**: B = 1000 DWI); arrows indicate the central pontine-demyelinization area

**Fig. 3:**
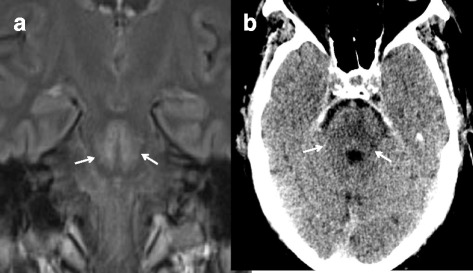
MRI image of the brain (**a** FLAIR cor) and cranial computed tomography image of the brain (**b** CE-cranial); arrows indicate the central pontine-demyelinization area

An T2-weighted magnetic resonance imaging (MRI) of the head (Fig. 2 A, B) obtained 3 days prior to hospitalization in an outpatient clinic demonstrated a hyperintense, symmetric, non-space occupying central pontine lesion. In diffusion-weighted MRI (Fig. 2 C and D, Fig. 3 A), the hyperintensity was confirmed. Ten days after admission, a cranial computed tomography scan (Fig. 3 B) revealed a hypodense symmetric central pontine lesion similar to the one demonstrated in the MRI, thereby confirming the diagnosis of a central pontine demyelinization syndrome.

Under conservative and physical therapy, both the overall condition and the glucose metabolism improved (Fig. 1). Both the balance and gait disturbance improved. However, the cognitive impairment and lack of orientation prevailed. After 26 days in hospital, the patient was discharged with an insulin regimen (intensified conventional therapy with insulin aspart: 14 units/d in euglycemia, otherwise correction, and insulin glargin 20 units/d.) and dulaglutide, a glucagon-like peptide-1 receptor agonist, and a vitamin B1 supplement. Upon discharge, the insulin therapy was temporarily applied by an outpatient ambulatory-care service. For further mobilization, a follow-up stay in an inpatient ambulatory rehabilitation clinic was performed 6 weeks after discharge. Three months after discharge, the patient greatly recovered. He was able to walk and to perform the insulin therapy without help. During the follow-up period after discharge and after rehabilitation, hypoglycemic episodes occurred. The family practitioner adjusted the insulin dose accordingly achieving a sufficient glycemic control.

## Discussion and conclusion

Here, we present a rare case of a central pontine myelinolysis after ingestion of an abnormally high amount of sugar over a short period of time. In addition, baseline glucose prior to this meal was likely to be elevated as shown by an extraordinarily elevated glycated hemoglobin A1c in absence of a prescribed insulin therapy. The concomitant hepatic encephalopathy and/or a alcoholism-associated dementia syndrome might have facilitated the inappropriate voluntary action leading to a severe hyperglycemia occurring in a close timely relationship with the central pontine demyelinization. In absence of any proven relevant hyponatremia, the resulting hyperglycemic episode is the likely cause for the central pontine myelinolysis in this patient. The mild hyponatremia on admission is regarded as a pseudohyponatremia due to the concomitant hyperglycemia and has been seen in a similar case in an acute setting [4], however, not in a subacute setting as shown in a case of a type-1-diabetic, non-ketoacidotic patient who temporarily stopped insulin treatment [5].

Interestingly, on admission, the level of consciousness appeared to be normal, a paresis was not found. Therefore, an urgent neurological exam was not mandated. The dysmetria of his right arm, the gait disturbance, dizziness, and vertigo were attributed to the hepatic encephalopathy first. By the 10th day in hospital or 16th day after glucose ingestion, both the coordination disturbances and the level of consciousness deteriorated, even though serum ammonia levels were lower. In absence of any other explanation, these neurological symptoms are attributed to the central pontine myelinolysis.

Hypothetically, osmotic myelinolysis may have been more widespread, i.e. not being restricted to the pons, thereby accounting for the impairment of consciousness in this case. The detailed mechanism, why symptoms are lagging behind the pathophysiolgical cue “hyperglycemia”, is unclear. Most likely, underlying conditions such as liver cirrhosis and encephalopathy may predispose to central pontine myelinolysis [9], and, hypothetically, affect the course of disease.

A decompensated liver cirrhosis may lead to shifts of water, when ascites is present. In addition, in cases of massive glucose ingestion, the liver capacity for glucose disposal may be limited. Hypothetically, self-control, psychological inhibition to cues may be reduced in Alcoholic Korsakoff’s Syndrome most likely due to structural or functional changes in the prefrontal cortex [10].

The current case report confirms the necessity to avoid rapid increases in serum osmolality due to hyperglycemia to prevent a central pontine demyelinization. To reach this goal, the use of sugar-sweetened beverages and the consumption of sugar should be restricted to less than 10% of the daily energy needs [11]. This minimum dietary target has not been met in this case. In addition, as proven by the current glycated hemoglobin A1c, repeat hyperglycemia existed at least for 6 weeks prior to hospitalisation. Specifically, early symptoms of a central pontine demyelinization may have been attributed to a hepatic encephalopathy, thereby delaying the diagnosis. Thus, this case shows that central pontine and/or cerebellar myelinolysis can be caused by sudden, severe, and sustained hyperglycemia, especially when another risk factor (in this case, liver cirrhosis) is present. New-onset, functional neurological deficits in the context of hyperglycemia should prompt for the consideration of this differential diagnosis. Clearly, in all diabetes patients, diabetes counseling and the control of insulin therapy, once initiated, are basic, measures to prevent central pontine myelinolysis.

## Abbreviations

eGFR: Estimated Glomerular Filtration Rate
HOMA-IR: Homeostasis Model Assessment of Insulin Resistance
KDIGO: Kidney Disease Improving Global Outcomes
MRI: Magnetic Resonance Imaging
